# Interferon-γ promotes abortion due to *Brucella *infection in pregnant mice

**DOI:** 10.1186/1471-2180-5-22

**Published:** 2005-05-04

**Authors:** Suk Kim, Dong Soo Lee, Kenta Watanabe, Hidefumi Furuoka, Hiroshi Suzuki, Masahisa Watarai

**Affiliations:** 1 Department of Applied Veterinary Science, Obihiro University of Agriculture and Veterinary Medicine, Obihiro, Hokkaido 080-8555, Japan; 2 Research Center for Protozoan Diseases, Obihiro University of Agriculture and Veterinary Medicine, Obihiro, Hokkaido 080-8555, Japan; 3 Department of Pathological Science, Obihiro University of Agriculture and Veterinary Medicine, Obihiro, Hokkaido 080-8555, Japan; 4 Department of Development and Medical Technology, Graduate School of Medicine, The University of Tokyo, Hongo, Tokyo 113-0033, Japan

## Abstract

**Background:**

The mechanisms of abortion induced by bacterial infection are largely unknown. In the present study, we investigated abortion induced by *Brucella abortus*, a causative agent of brucellosis and facultative intracellular pathogen, in a mouse model.

**Results:**

High rates of abortion were observed for bacterial infection on day 4.5 of gestation, but not for other days. Regardless of whether fetuses were aborted or stayed alive, the transmission of bacteria into the fetus and bacterial replication in the placenta were observed. There was a higher degree of bacterial colonization in the placenta than in other organs and many bacteria were detected in trophoblast giant cells in the placenta. Intracellular growth-defective *virB4 *mutant and attenuated vaccine strain S19 did not induce abortion. In the case of abortion, around day 7.5 of gestation (period of placental development), transient induction of IFN-γ production was observed for infection by the wild type strain, but not by the *virB4 *mutant and S19. Neutralization of IFN-γ, whose production was induced by infection with *B. abortus*, served to prevent abortion.

**Conclusion:**

These results indicate that abortion induced by *B. abortus *infection is a result of transient IFN-γ production during the period of placental development.

## Background

Brucellosis is a widespread and economically important infectious disease of animals and humans caused by members of the genus *Brucella*. *Brucella *spp. are small gram-negative, facultative intracellular pathogens that cause abortion, retained placenta and infertility in numerous domestic and wild mammals, and a disease known as undulant fever in humans [1,2]. Infection in humans is almost exclusively due to zoonosis, either through direct contact with infected animals or from contaminated dairy products [3]. The mouse model, particularly that using the unpregnant mouse, has been used extensively to study some aspects of the pathogenesis of brucellosis [4]. While brucellosis is known to primarily affect the reproductive tract in the natural host and has been much studied, little is known regarding the cellular and molecular mechanisms of *Brucella *infection in the pregnant mouse [5]. There have been no studies on the induction of abortion by *Brucella *infection in the pregnant mouse.

A key aspect of the virulence of *Brucella *is its ability to proliferate within professional and non-professional phagocytic host cells, thereby successfully bypassing the bactericidal actions of phagocytes, which is thought to explain its virulence and ability to cause chronic infections [6,7]. The molecular mechanisms and genetic basis for its intracellular survival and replication, however, are not understood completely. Recent studies with non-professional phagocyte HeLa cells show that *Brucella *inhibits phagosome-lysosome fusion and transits through an intracellular compartment that resembles autophagosomes. Bacteria replicate in a different compartment, containing protein markers normally associated with the endoplasmic reticulum, as shown by confocal microscopy and immunogold electron microscopy [8-10].

Pregnancy leads to a generalized suppression of the adaptive immune system, typified by significantly decreased cell-mediated immunity and reduced T helper cell (Th) 1 responsiveness [11-13]. This immunosuppressed state prevents maternal rejection of the fetus but has the unfortunate consequence of increasing maternal susceptibility to certain infectious agents [14,15]. Immunity against *B. abortus *is principally mediated by cellular immune responses since it is an intracellular pathogen, and involves antigen-specific T-cell activation of CD4 and CD8 T cells and humoral responses. Protection of the host against *B. abortus *infection is thought to be mediated primarily by a Th1 type of immune response [16]. For many other intracellular bacterial and protozoan pathogens, it has been shown that interferon-γ (IFN-γ) is an important component of Th1 immune responses and contributes to control through its ability to stimulate macrophages to kill more microbes. The role of IFN-γ in the control of *B. abortus *infections has been demonstrated by supplementing BALB/c mice with recombinant IFN-γ, when such treatment resulted in a 10-fold decrease in the number of bacteria at 1 week after infection [17]. It has also been shown that neutralizing endogenous IFN-γ by administering anti-IFN-γ monoclonal antibodies results in a decrease in control [16]. Despite these results, however, the role of cytokines in the control of *B. abortus *infection in the pregnant mouse is still unknown.

In the present study, we investigated the pathogenesis of *B. abortus *in the pregnant mouse and established a mouse model for abortion induced by *B. abortus *infection. We noted that IFN-γ plays an important role in the induction of abortion by *B. abortus *infection.

## Results

### *B. abortus *infection causes abortion in pregnant mice

**Figure 1:**
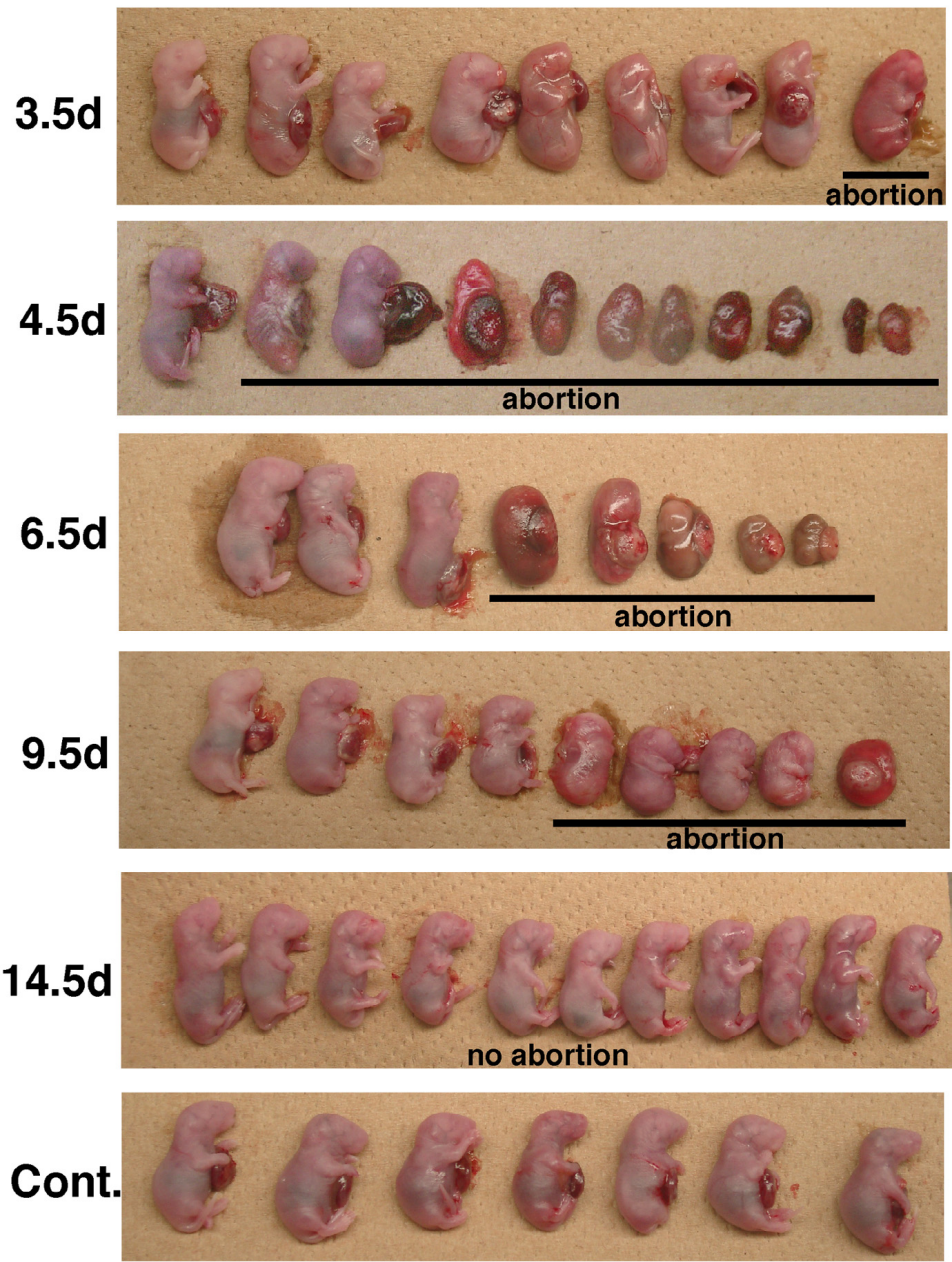
*B. abortus *infection causes abortion in pregnant mice. Groups of four pregnant mice were infected with bacteria intraperitoneally at each gestation time point. On day 18.5 of gestation, their fetuses were removed. Fetuses were determined to be alive if there was a heartbeat, and dead if there was no heartbeat. Fetuses underlined were aborted.

In order to construct a mouse model of abortion due to *B. abortus *infection, the numbers of aborted fetuses in infected mice were counted on day 18.5 of gestation. For infection with wild type *B. abortus *on day 4.5 of gestation, all fetuses, except for those of 1 mouse (No. 2), were aborted (98.4% abortion) (Table 1) (Fig. 1). For infection on days 3.5, 6.5 and 9.5 of gestation, the majority of fetuses were still alive (28.1–37.3% abortion), and no abortion was observed for infection on day 14.5 of gestation (Table 1) (Fig. 1). The endpoint number of delivered mice in pregnant mice infected on day 4.5 of gestation was 2, but that in pregnant mice infected on the other days of gestation or in uninfected pregnant mice was 16. Replicating bacteria were observed in both aborted fetuses and those that lived, and there were no significant differences in bacterial infection rates between live (average 3.43 log CFU) and aborted fetuses (average 4.10 log CFU) (Table 1). These experiments were performed at least three times with the same results.

### *B. abortus *infection predominantly in placenta

**Figure 2:**
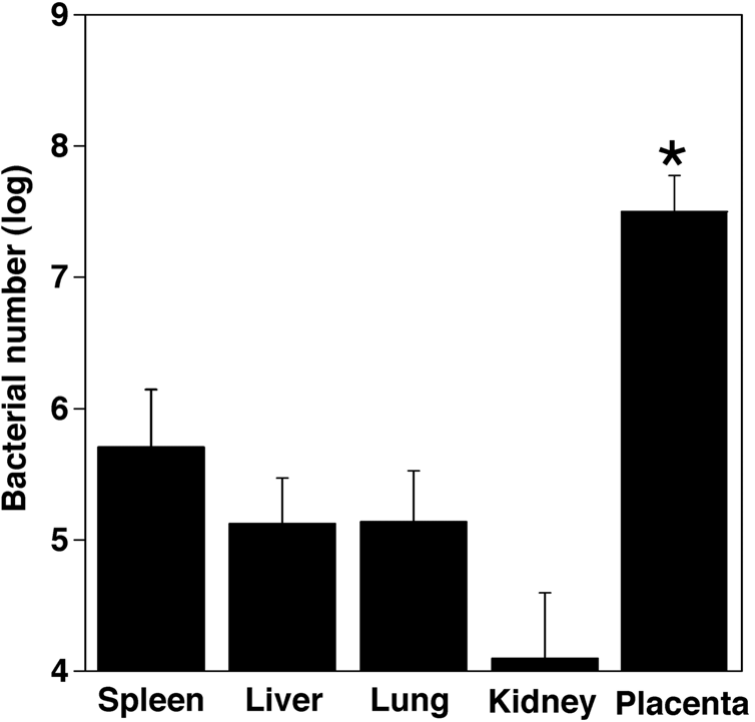
*B. abortus *infection predominantly in placenta. Groups of four pregnant mice were infected with bacteria intraperitoneally on day 6.5 of gestation. On day 18.5 of gestation, their placenta, spleen, liver, lungs, and kidneys were removed and homogenized in saline. Tissue homogenates were serially diluted with PBS and plated on Brucella agar to count the number of CFU in each organ. Statistically significant differences bacterial numbers between placenta and spleen are indicated by asterisks (*, P < 0.01).

**Figure 3:**
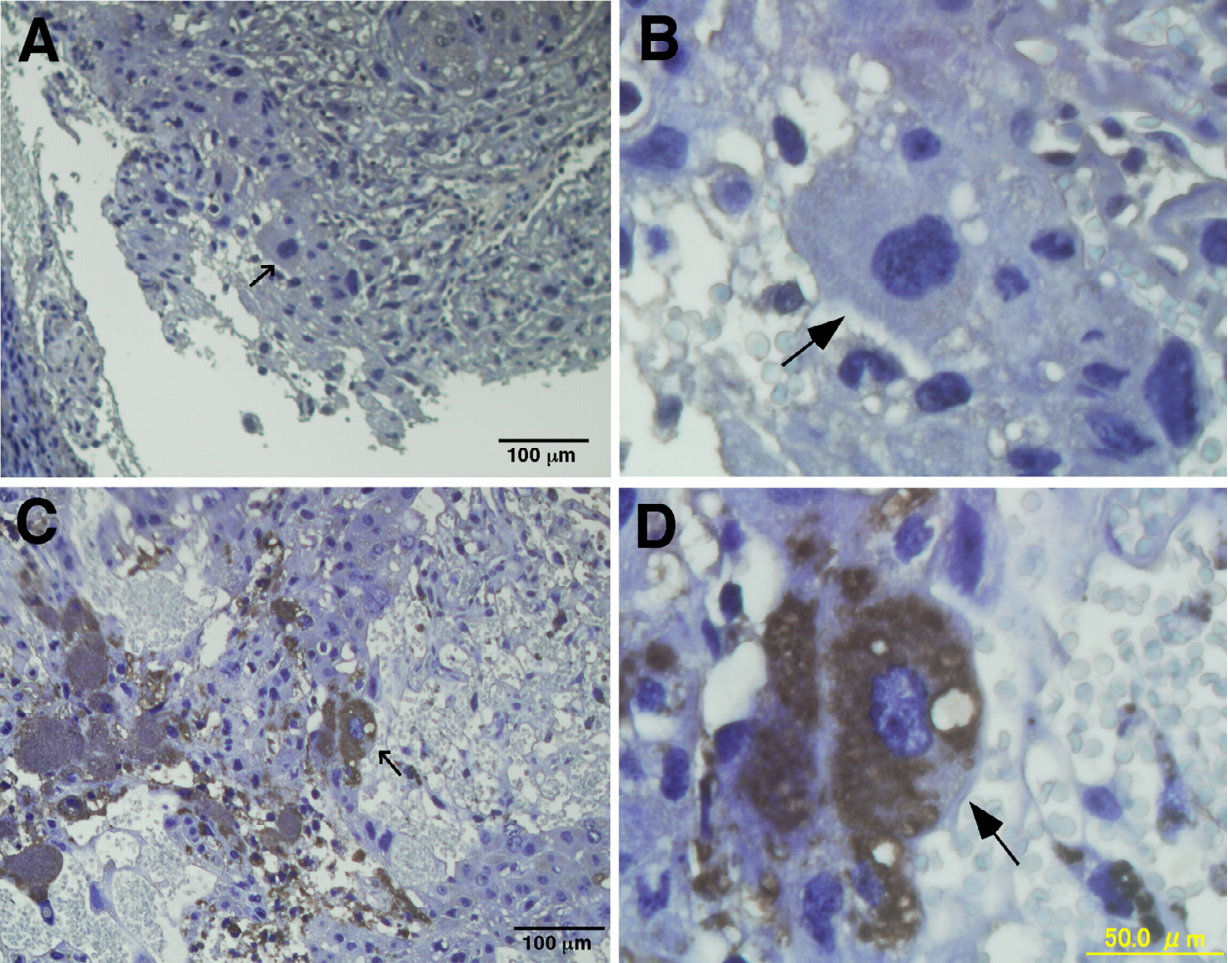
*B. abortus *predominantly invades into trophoblast giant cells in placenta. Placentas not used for bacterial culture were fixed in situ within uteri in 10% neutral buffered formalin and processed routinely for histologic examination and scoring using Meyer's hematoxylin stain. Specific labeling of *B. abortus *in placental sections was performed using the Dako EnVision System with anti-*B. abortus *rabbit serum and replicated bacteria are shown in brown. Normal (A and B) and infected placenta (C and D) are shown. Panels (B) and (D) indicate magnified images in panels (A) and (B). Arrows show trophoblast giant cells.

It is well known that the placenta plays an integral role in the pathogenesis of congenital infections. However, the placental factors controlling these processes are not completely understood. To investigate the role of the placenta in *B. abortus *infection, we examined the placental colonization of the wild type strain. Replicating bacteria were observed in both the placentas of live and aborted fetuses, and the difference between the bacterial growth rate in the placenta of live (average 7.12 log CFU) and aborted fetuses (average 7.17 log CFU) was not significant (Table 1). The bacterial growth rate in the spleen was lower than that in the placenta in all cases (Table 1). In order to check whether *B. abortus *colonizes the placenta predominantly, bacterial growth in the liver, lung and kidney were measured in addition to that in the spleen and placenta for infection on day 6.5 of gestation. As expected, colonization by *B. abortus *was much greater in the placenta than in the other organs (Fig. 2). Immunostaining of bacteria infected placenta specimens revealed that bacteria were present in numerous trophoblast giant cells (TGC) along the periphery of the placenta. Bacteria were also observed in neutrophils associated with the regressing deciduas capsularis or free within the newly formed uterine lumen adjacent to this region of the placenta (Fig. 3). Replicating bacteria were observed in TGC in both the placentas of live and aborted fetuses (data not shown).

### Type IV secretion system of *B. abortus *contributes to abortion indirectly

As the internalization and intracellular replication of *B. abortus *are controlled by the type IV secretion system [18,19], on day 4.5 of gestation, pregnant mice were infected with the *virB4 *mutant, which does not have the ability of intracellular replication [20,21]. The inoculation of pregnant mice with the *virB4 *mutant did not induce abortion. Bacterial growth, however, was observed in the fetus, placenta and spleen (Table 2). We also observed that attenuated live vaccine strain S19 did not induce abortion (Table 2), even though in contrast to the *virB4 *mutant it has been seen to have replication capability in macrophages [22,23]. These results suggest that the type IV secretion system may not contribute to abortion directly when mice were infected with *B. abortus*, because S19 has intact *virB *genes.

### Transient increase in IFN-γ in pregnant mice induced by *B. abortus *infection

**Figure 4:**
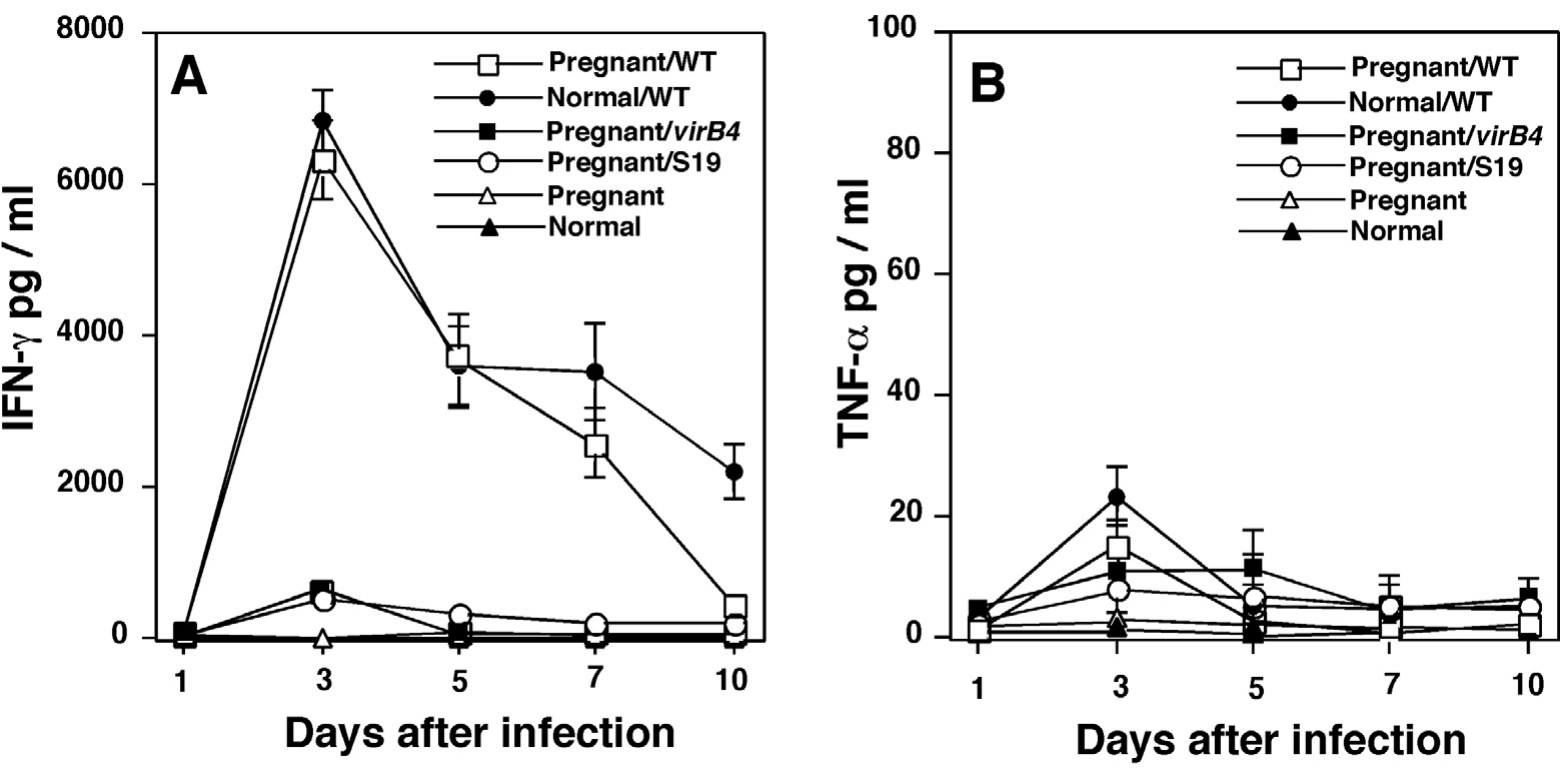
Transient increase in IFN-γ in pregnant mice induced by *B. abortus *infection. The IFN-γ (A), and TNF-α (B) serum levels were measured in each mouse by ELISA at the indicated numbers of days after infection. The mean and SE for groups of five mice are shown.

Though fetal tissues possess paternal antigens, the fetus is thought to be insulated from rejection by the maternal immune system. The precise cellular and molecular mechanisms underlying the maintenance of pregnancy and the induction of abortion, however, remain unclear. As recent studies have shown that Th1 cytokines, such as interferon-γ (IFN-γ) and tumor necrosis factor α (TNF-α), induce abortion [24], we decided to determine if IFN-γ and TNF-α contribute to the induction of abortion by *B. abortus *infection and measured the production of IFN-γ and TNF-α by ELISA in pregnant mice infected on day 4.5 of gestation. A large amount of induced IFN-γ production was observed at 3 days after infection with the wild type strain (day 7.5 of gestation) but this production of IFN-γ then decreased rapidly (Fig. 4A). A similar pattern of IFN-γ induction was observed after infecting unpregnant mice with the wild type strain. However, neither the *virB4 *mutant nor the attenuated live vaccine strain S19 induced IFN-γ production (Fig. 4A). In addition, no significant induction of TNF-α production was observed in the infected pregnant mice (Fig. 4B). These results imply that IFN-γ contributes to abortion due to *B. abortus *infection, and increased production of IFN-γ around day 7.5 of gestation may be critical, but that TNF-α is not significantly involved.

### Preventing abortion by neutralizing IFN-γ production

**Figure 5:**
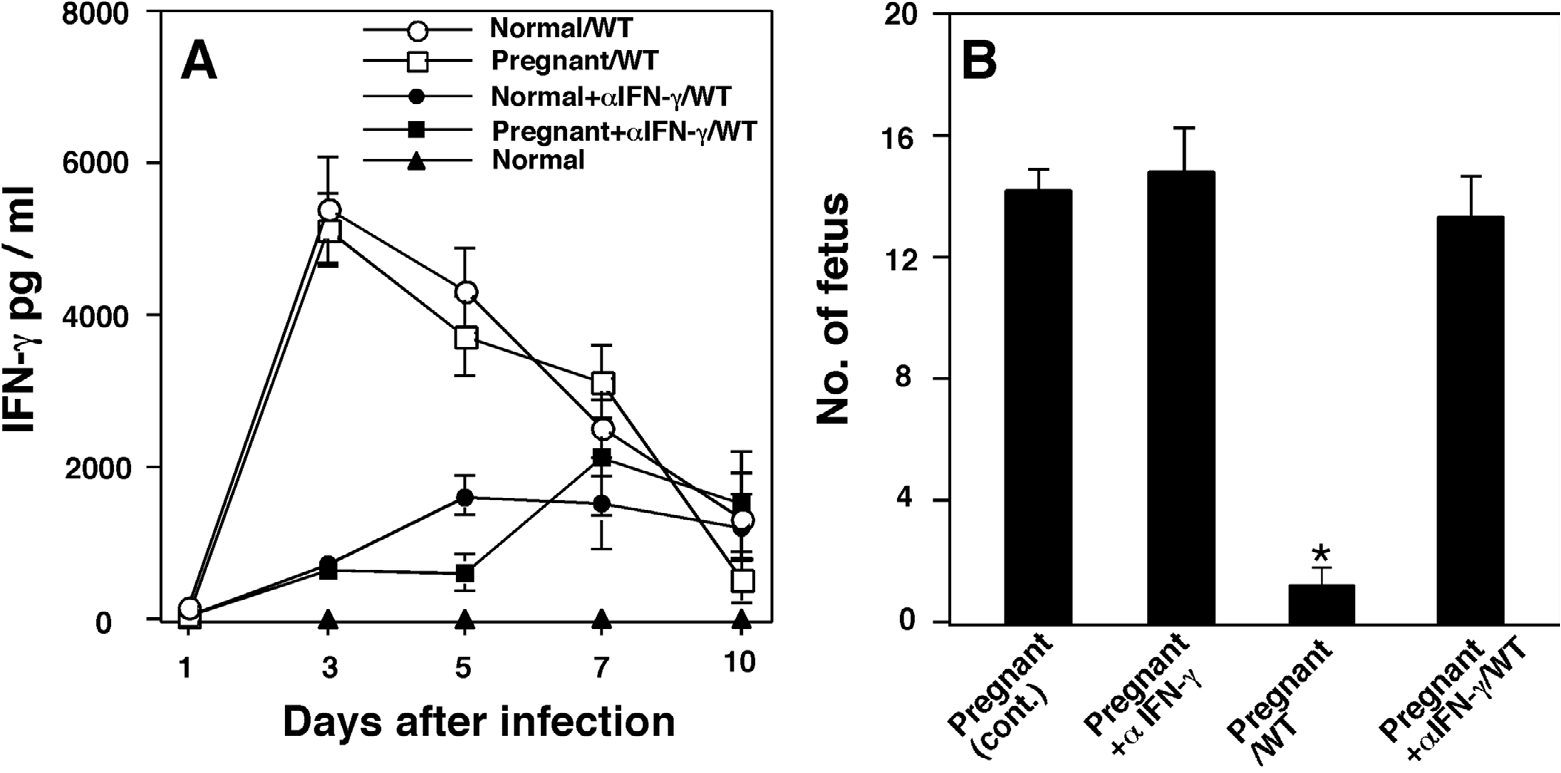
Preventing abortion by neutralizing IFN-γ. IFN-γ was neutralized in mice by administering anti-mouse IFN-γ monoclonal antibody (clone HB170) *in vivo *using 1 mg of antibody in a volume of 0.5 ml intraperitoneally 24 h before infection. Control mice were given 1 mg of normal rat IgG in 0.5 ml according to the same injection schedule as the corresponding anti-IFN-γ monoclonal antibody treatment groups. (A) IFN-γ in serum measured by ELISA. The mean and SE for groups of five mice are shown (B) Number of fetuses. Statistically significant differences between the untreated control and antibody treated mice are indicated by asterisks (*, P < 0.01).

To determine if abortion is prevented by neutralizing the IFN-γ produced as a result of bacterial infection, pregnant mice were inoculated with monoclonal anti-IFN-γ antibody 1 day before infection with *B. abortus*, which was done on day 4.5 of gestation. Thereafter, the serum levels of IFN-γ and abortion rates were recorded. A large amount of induced IFN-γ production was observed 3 days after infection (day 7.5 of gestation) in the pregnant mice and unpregnant control for mice without antibody, and this production was inhibited in mice inoculated monoclonal anti-IFN-γ antibody for both pregnant mice and the unpregnant control (Fig. 5A). Also, while abortions were observed in the untreated pregnant mice as before, none were seen in pregnant mice inoculated with anti-IFN-γ antibody (Fig. 5B). These results indicate that IFN-γ production contributes to abortion due to *B. abortus *infection in pregnant mice.

## Discussion

In the present study we investigated abortion induced by *B. abortus *infection in a mouse model. In a previous study, Tobias *et al*. observed no abortion in pregnant BALB/c mice infected with *B. abortus *virulent strain 2308 on day 9 of gestation, but the bacteria multiplied in the placenta and could be identified in trophoblast giant cells (TGC) [5]. In our mouse model, high abortion rates were seen with bacterial infection on day 4.5 of gestation and lower rates were observed when infection was on day 9.5 of gestation. It thus seems that the day of infection during gestation is critical for induction of abortion in mouse models, and this may explain the difference between our results and those of the previous study. Through inoculation with wild type *B. abortus *on day 4.5 of gestation, an increase in the production of IFN-γ was induced around day 7.5 of gestation. It is thought that the serum IFN-γ level in this period of gestation is relevant to the maintenance of the fetus in the pregnant mice and when the amount of IFN-γ increases during this critical period of pregnancy, abortion may occur [13]. And we observed that abortion due to bacterial infection was indeed prevented when the amount of IFN-γ decreased during the critical period due to neutralization with anti-IFN-γ monoclonal antibody. Cytokine profiles have been observed to have physiological implications for pregnancy, perhaps the most profound being the detrimental affect of T helper cell (Th) 1 cytokines such as IFN-γ and TNF-α, and associated cells and mediators. Thus, natural killer (NK) cells, IFN-γ, TNF-α and nitric oxide (NO) can be deleterious to the well-being of the fetoplacental unit, and capable of inducing abortion and fetal resorption [13]. Our results indicated that the Th1 cytokine IFN-γ play a key role in pregnancy.

Our study and that of Tobias *et al*. showed that *B. abortus *replicated preferentially in the TGC in the placenta [5]. The mechanism for this is unclear. Erythritol, a sugar alcohol synthesized in the ungulate placenta and known to stimulate the growth of virulent strains of *B. abortus*, has been credited with the preferential localization of this bacterium within the placenta of ruminants [25,26]. Although very low concentrations of erythritol are found in the placentas of rodents [25], *B. abortus *will localize and proliferate preferentially in their placenta [[5,27], this study]. While the growth of attenuated vaccine strain S19 was inhibited by erythritol, but its growth was still preferentially localized in the ruminant placenta [26]. Two placental regions can be recognized: a junctional zone and a labyrinth zone. The junctional zone is comprised of stem cells and three differentiated cell types: TGC, spongiotrophoblast cells, and glycogen cells. TGC arise by endoreduplication, are situated at the maternal-placental interface, and are one of the major endocrine cells of the placenta [28]. TGC are polyploidy cells that play a crucial role during implantation and in remodeling of the embryonic cavity, avoidance of maternal immune rejection and promotion of maternal blood flow to the implantation site [29]. TGC should have been hijacked by *B. abortus *so that cell functions were not exhibited completely, this leading to abortion due to inhibition of implantation and placental development. However, we observed replicating bacteria in the TGC in both placentas of live and aborted fetuses. TGC, spongiotrophoblasts and the labyrinth zone are established from day 8.5 to 10.5 of gestation and comprise the definitive placenta that persists until the end of gestation [29]. Therefore, abortion-inducing factors should act just before day 8.5 of gestation, adding to bacterial infection in the TGC. As abortion occurs in pregnant mice when IFN-γ production is induced by the bacterial infection around day 7.5 of gestation, IFN-γ may influence the development of TGC. In this regard, IFN-γ has been shown to inhibit the cells of the trophoblast lineage outgrowth and trophoblast cell invasion to be accelerated in mice with genetic deficiency in the IFN-γ or IFN-γ receptor [30]. Presumably, IFN-γ production induced by bacterial infection inhibits the development of TGC and *B. abortus *invades the remaining small number of TGC around day 7.5 of gestation. TGC completely lose all of their function due to the effect of IFN-γ and bacterial replication inside them.

Natural killer (NK) cells are large granular lymphocytes found in peripheral blood and also in the maternal decidua during pregnancy. The actions of NK cells on trophoblast lineage cells are likely mediated by NK cell secretory products, including cytokines, and may be direct or indirect. Uterine NK cells produce several cytokines and are the primary source of INF-γ in the metrial grand [30,31]. IFN-γ has been implicated as a major mediator of uterine NK cell function during pregnancy [32,33]. Trophoblast cells are among a variety of different IFN-γ targets, and *in vitro *trophoblast cell differentiation, survival, and outgrowth are affected by IFN-γ [30,34]. IFN-γ is a crucial cytokine for *Brucella *immunity [35,36]. The major function of IFN-γ in *Brucella *immunity is the stimulation of bactericidal activity in macrophages, host cells of *Brucella*. However, the function of IFN-γ is more diverse than the induction of bactericidal function and includes the stimulation of antigen presentation through class I and class II MHC molecules, the orchestration of leukocyte-endothelium interactions, the effects on cell proliferation and apoptosis, as well as stimulation and repression of a variety of genes whose functional significance remains obscure [37]. Though a previous study found that little role for NK cells in controlling brucellosis in unpregnant mice when NK cells were depleted by administering monoclonal antibody despite the activation of these cells following infection [38], NK cells may play a role in controlling brucellosis in pregnant mice because the modulation of host immune responses differs between pregnant mice and unpregnant normal mice [39].

The *virB4 *mutant, which is defective intracellular growth, did not cause abortion in pregnant mice, suggesting that the type IV secretion system of *B. abortus *contributes to abortion induced by bacterial infection. Our results also showed that the *virB4 *mutant replicated in the placenta. Presumably, *virB4 *mutant replicates extracellulary in the placenta due to maternal immunosuppression during pregnancy [40]. Wild type *B. abortus *internalizes into macrophages by swimming on the cell surface with generalized membrane ruffling for several minutes, a process termed "swimming internalization", after which the bacteria are enclosed by macropinosomes [41]. In this period, the phagosomal membrane continues to maintain a dynamic state. Lipid raft-associated molecules, such as glycosylphosphatidylinositol (GPI)-anchored proteins, GM1 ganglioside and cholesterol, have been shown to be selectively incorporated into macropinosomes containing *B. abortus*. While late endosomal marker lysosomal-associated membrane protein (LAMP)-1 and host cell transmembrane proteins are excluded from the macropinosomes. The disruption of lipid rafts on macrophages markedly inhibits the type IV secretion system-dependent macropinocytosis and intracellular replication [21]. Thus, *B. abortus *may internalize into TGC by lipid rafts-dependent pathway like that of macrophages, and such internalization and intracellular replication of *B. abortus *may contribute to induction of IFN-γ in pregnant mice. Our recent study showed that *B. abortus *Hsp60 (a member of the GroEL family of chaperonins) was secreted on the bacterial surface through the type IV system and that it can interact with the cellular prion protein (PrP^C^) on macrophages [41]. This interaction is important for establishment of *B. abortus *infection. As prion protein accumulates in the placenta [42], PrP^C ^may be involved in the internalization of *B. abortus *into TGC.

Many vertically transmitted pathogens are detrimental to the welfare of livestock. Among these are *Listeria monocytogenes*, *Chlamydophila abortus*, *Toxoplasma gondii*, *Neospora caninum*, bovine viral diarrhoea virus (BVDV) and Border disease virus (BDV). These diverse organisms, ranging from bacteria to apicomplexan protozoa and RNA viruses, share some common features. They give rise to mild and often inapparent disease in non-pregnant animals but cause abortion in ruminants and also in human beings in some cases. However, the disease processes and mechanisms by which they cause abortion are different [39]. IFN-γ and TNF-α are central to host immunity against listeriosis, but can compromise fetal survival [13]. Pregnant mice mount lower levels of protective Th 1 cytokines and are unable to eliminate the pathogen. This leads to severe necrotizing hepatitis and bacteria-induced placental necrosis, increasing the incidence of postimplantation loss and a poor pregnancy response [43].

## Conclusion

*B. abortus *appears to be able to exploit the very precise regulatory processes that control immune responsiveness during pregnancy. Our observations suggest that the generation of inflammatory responses in the maternal periphery and at the maternofetal interface are detrimental for fetal survival and this has important implications for pathogen control during pregnancy. Therefore, vaccination strategies to control *B. abortus *have to be carefully designed and implemented appropriately to prevent the generation of inappropriate inflammatory responses that might affect the outcome of pregnancy. Thus, using our mice model, we should investigate the mechanism of abortion due to *B. abortus *infection in more detail and develop on methods for the prevention of abortion that are safer than current control programs.

## Methods

### Bacterial strains and mice

All *B. abortus *derivatives were from 544 (ATCC23448) smooth virulent *B. abrotus *biovar 1 strains. Ba598 (544Δ*virB4*) and *B. abortus *vaccine strain S19 were used in this study [20]. *B. abortus *strains were maintained as frozen glycerol stocks and cultured on Brucella broth (Becton Dickinson) or Brucella broth containing 1.5% agar.

### Mice

Six to ten-week-old ICR female mice were individually mated to 6- to 10-week-old ICR male mice. The parent mice were obtained from CLEA Japan. Day 0.5 of gestation was the day the vaginal plug was observed. The normal gestational time for these mice is 19 days.

### Virulence in pregnant mice

Groups of four or five pregnant mice were infected intraperitoneally with approximately 10^4 ^CFU of brucellae in 0.1 ml saline at the indicated days of gestation. On day 18.5 of gestation, their fetus, placenta, spleen, liver, lungs, and kidneys were removed and homogenized in saline. Tissue homogenates were serially diluted with PBS and plated on Brucella agar to count the number of CFU in each spleen. Fetuses were determined to be alive if there was a heartbeat, and dead if there was no heartbeat.

### Histology

Placentas not used for bacterial culture were fixed in situ within uteri in 10% neutral buffered formalin and processed routinely for histologic examination and scoring using Meyer's hematoxylin stain. Specific labeling of *B. abortus *in placental sections was performed using the Dako EnVision System with anti-*B. abortus *rabbit serum [21].

### Cytokine measurement

The level of serum IFN-γ and TNF-α were measured for infected and uninfected virgin and pregnant ICR mice. Groups of five mice were infected intraperitoneally with approximately 10^4 ^CFU of brucellae in 0.1 ml saline on day 4.5 of gestation, and blood was collected at 1, 3, 5, 7 or 10 days after infection. Blood was collected at the same time periods for uninfected mice. On day 18.5 of gestation, their uteruses were removed and a judgment was made as to whether mice were pregnant or not. Serum levels of IFN-γ and TNF-α were measured with an enzyme linked immunosorbent assay (ELISA) kit (PIERCE Endogen) according to the instructions of the manufacturer.

### *In vivo *depletion of IFN-γ

IFN-γ was neutralized in the mice by administering anti-mouse IFN-γ monoclonal antibody (clone HB170) *in vivo *using 1 mg of antibody in a volume of 0.5 ml intraperitoneally 24 h before infection. Control mice were given 1 mg of normal rat IgG in 0.5 ml according to the same injection schedule as the corresponding anti-IFN-γ monoclonal antibody treatment groups. Bacterial infection was done as described above. Blood was collected at 1, 3, 5, 7 or 10 days after infection, and the serum levels of IFN-γ and TNF-α were measured with an ELISA kit as described above. On day 18.5 of gestation, their uteruses were removed and a judgment was made as to whether mice were pregnant or not. Fetuses were determined to be alive if there was a heartbeat, and dead if there was no heartbeat.

### Statistical analysis

All statistical analysis were calculated using Student *t *test.

## Authors' contributions

SK carried out all virulence assays and analysis of the data. DSL and KW carried out mice mating and maintenances. HF carried out pathological experiments. HS and MW conceived of the study and participated in its design and coordination.

## Figures and Tables

**Table 1:** *B. abortus *infection in pregnant mice.

		Fetus						Placenta				Spleen	
				
Days of gestation	Mouse No.	Alive			Aborted			Alive		Aborted			
							
		No.	Weight (g)*	Bacterial No. (log)*	No.	Weight (g)*	Bacterial No. (log)*	Weight (g)*	Bacterial No. (log)*	Weight (g)*	Bacterial No. (log)*	Weight (g)	Bacterial No. (log)
3.5	1	14	1.21 (±0.10)	3.82 (± 0.36)	1	0.99	3.84	0.12 (±0.02)	7.48 (±0.24)	0.13	7.51	0.65	5.93
	2	0	-	-	14**	-	-	-	-	-	-	0.46	6.11
	3	11	1.32 (±0.13)	3.11 (±0.24)	1	1.13	3.27	0.15 (±0.01)	7.52 (±0.35)	0.14	7.39	0.67	6.12
	4	16	1.42 (±0.11)	2.18 (±0.13)	0	-	-	0.13 (±0.02)	7.55 (±0.41)	-	-	0.32	5.62

4.5	1	0	-	-	16**	-	-	-	-	-	-	0.55	5.30
	2	1	1.33	3.32	14	0.82 (±0.26)	3.56 (±0.27)	0.14	7.42	0.18 (±0.02)	5.36 (±0.23)	0.82	6.12
	3	0	-	-	17**	-	-	-	-	-	-	0.51	5.14
	4	0	-	-	15**	-	-	-	-	-	-	0.92	6.11

6.5	1	12	1.12 (±0.14)	4.21 (±0.31)	5	1.00 (±0.22)	4.18 (±0.20)	0.15 (±0.01)	7.35 (±0.24)	0.11 (±0.01)	7.18 (±0.22)	0.39	6.12
	2	16	1.42 (±0.21)	3.23 (±0.33)	2	1.00 (±0.14)	4.13 (±0.31)	0.12 (±0.02)	4.72 (±0.32)	0.09 (±0.02)	7.33 (±0.41)	0.18	5.41
	3	0	-	-	15	0.61 (±0.18)	5.22 (±0.27)	-	-	0.11 (±0.01)	7.24 (±0.38)	1.22	6.43
	4	12	1.38 (+0.10)	4.14 (+0.29)	1	0.95	4.36	0.14 (±0.02)	7.52 (±0.31)	0.11	7.43	0.47	6.19

9.5	1	0	-	-	14**	-	-	-	-	-	-	0.70	6.12
	2	10	1.23 (±0.16)	3.53 (±0.14)	5	0.93 (±0.10)	4.11 (±0.24)	0.11 (±0.01)	7.52 (±0.32)	0.08 (±0.02)	7.55 (±0.31)	0.65	6.13
	3	15	1.02 (±0.08)	4.29 (±0.21)	3	1.02 (±0.21)	4.23 (±0.19)	0.11 (±0.01)	7.54 (±0.26)	0.12 (±0.02)	7.53 (±0.27)	0.46	6.11
	4	12	1.43 (±0.19)	3.14 (±0.16)	0	-	-	0.14 (±0.02)	7.35 (±0.38)	-	-	0.26	5.19

14.5	1	12	1.61 (±0.18)	4.17 (±0.19)	0	-	-	0.11 (±0.01)	7.22 (±0.29)	-	-	0.18	6.15
	2	14	1.32 (±0.12)	3.42 (±0.21)	0	-	-	0.11 (±0.01)	7.11 (±0.31)	-	-	0.29	6.81
	3	10	1.72 (±0.21)	2.24 (±0.14)	0	-	-	0.14 (±0.01)	6.13 (±0.27)	-	-	0.14	5.42
	4	11	1.43 (±0.14)	3.17 (±0.17)	0	-	-	0.11 (±0.02)	7.21 (±0.39)	-	-	0.26	6.91

Cont.	1	15	1.45 (±0.11)	-	-	-	-	0.13 (±0.01)	-	-	-	0.20	-
	2	11	1.59 (±0.16)	-	-	-	-	0.12 (±0.01)	-	-	-	0.14	-
	3	14	1.63 (±0.12)	-	-	-	-	0.13 (±0.02)	-	-	-	0.15	-

*Average number of fetuses or placentas.

**Number of mummifications.

**Table 2:** Infection of pregnant mice with *B. abortus virB4 *mutant or vaccine strain S19 on day 4.5 of gestation.

		Fetus						Placenta				Spleen	
				
Strain	Mouse No.	Alive			Aborted			Alive		Aborted			
							
		No.	Weight (g)*	Bacterial No. (log)*	No.	Weight (g)*	Bacterial No. (log)*	Weight (g)*	Bacterial No. (log)*	Weight (g)*	Bacterial No. (log)*	Weight (g)	Bacterial No. (log)
*virB4 *mutant	1	15	1.51 (±0.21)	3.25 (±0.16)	0	-	-	0.11 (±0.01)	7.51 (±0.26)	-	-	0.16	5.41
	2	16	1.43 (±0.18)	3.24 (±0.15)	0	-	-	0.14 (±0.01)	5.15 (±0.27)	-	-	0.20	5.38
	3	16	1.62 (±0.13)	1.14 (±0.08)	0	-	-	0.12 (±0.02)	5.52 (±0.34)	-	-	0.15	4.80
	4	13	1.51 (±0.15)	2.52 (±0.12)	0	-	-	0.12 (±0.02)	7.25 (±0.36)	-	-	0.21	5.61
	5	14	1.72 (±0.17)	3.64 (±0.24)	0	-	-	0.14 (±0.01)	7.48 (±0.33)	-	-	0.18	5.41

S19	1	14	1.43 (±0.14))	2.54 (±0.24)	0	-	-	0.13 (±0.01)	7.52 (±0.27)	-	-	0.50	7.12
	2	14	1.45 (±0.18)	2.24 (±0.20)	0	-	-	0.10 (±0.01)	7.51 (±0.35)	-	-	0.20	6.56
	3	15	1.35 (±0.14)	3.23 (±0.16)	0	-	-	0.10 (±0.02)	7.58 (±0.41)	-	-	0.21	6.63
	4	12	1.34 (±0.17)	3.17 (±0.27)	1	1.28	3.23	0.11 (±0.01)	7.64 (±0.21)	0.10	7.39	0.26	6.53
	5	14	1.35 (±0.12)	3.26 (±0.12)	0	-	-	0.10 (±0.01)	7.62 (±0.29)	-	-	0.24	6.96

*Average number of fetuses or placentas.
